# The Accuracy of GP Referrals into Manchester Royal Eye Hospital Orthoptic Department

**DOI:** 10.22599/bioj.168

**Published:** 2021-06-10

**Authors:** Martha Waters, Rachel Clarke, Laura England, Anna O’Connor

**Affiliations:** 1 Manchester Foundation Trust, GB; 2 University of Liverpool, GB

**Keywords:** referral, orthoptic, general practitioner, accuracy

## Abstract

**Background:**

There is little research that examines the accuracy of paediatric referrals into secondary and tertiary care, particularly those from general practicioners (GPs) to ophthalmology and orthoptic departments. Inaccurate referrals could have a detrimental effect on service delivery and NHS funding as well as patient experience. Available evidence shows GP referral accuracy to range between 39% and 90% across different areas of medicine with accuracy of GP referral to ophthalmology between 56% and 66%.

**Methods:**

A retrospective case note analysis was carried out on 99 case notes to examine the accuracy of paediatric GP referrals (including those via the community optometrist) into the Orthoptic Department at Manchester Royal Eye Hospital (MREH).

**Results:**

GP referral accuracy was found to be 63% for strabismus cases, 50% for reduced vision/amblyopia cases, 45% for NAD cases, 100% of nystagmus cases and 92% of “other” cases. GPs were significantly less accurate than community optometrists (p = 0.01). Referrals from GPs alone had an accuracy rate of 65% compared to 87% of GP referral via community optometrist. Accuracy of referral appeared to improve with age, however this was not found to be statistically significant (p = 0.06).

**Conclusion:**

This study found orthoptic referral accuracy for GPs in Manchester to be similar to other areas of medicine. While acceptable compared to other areas of medicine, improving referral accuracy is essential to improve NHS spending, service delivery, and patient experience. To aid with this the aim is to design and implement a virtual training package that focuses on detection of strabismus to improve referral accuracy.

## Introduction

General practitioners are responsible for onward referral of a wide range of medical conditions to secondary and tertiary care. Inaccurate referrals could have a detrimental effect on service delivery and NHS funds (Mashta 2010) as well as patient experience. Therefore it is essential that primary healthcare workers endeavour to streamline this process with the aim to increase referral accuracy. Evidence shows that accuracy of referrals from GPs varies between 39% and 89.8% across different areas of medicine (Elwyn 1994; Blomgren et al. 2003; Ahmed et al. 2014; Gormley et al. 2003).

Within ophthalmology there is little research that examines the accuracy of referrals from GPs. The accuracy of ophthalmological diagnosis from the referral was found to be 66% in 1990 (Wang et al. 1990) and 56% in 2016 (Davey et al. 2016). Jones et al. (1990), criticised GP referrals into ophthalmology for lacking detail and evidence of ocular examination. In addition, GP referral letters compared unfavourably to optometrist referrals and the authors concluded that undergraduate education in ophthalmology was inadequate with more curricular time required. While this study is 30 years old, a more recent study found length of ophthalmology education to be unchanged, with an average of only 7.6 days of ophthalmology training at undergraduate level (Baylis et al. 2011), making the conclusions valid to current knowledge.

As GPs are required to have knowledge in all areas of medicine, it is unreasonable to expect them to be experts in relation to common orthoptic conditions such as strabismus and amblyopia. However, any support that can be offered to increase the accuracy of referrals may reduce the number of appointments required, reduce waiting lists, and therefore improve patient experience. A possible reason for inaccurate paediatric referrals could be that paediatrics are a patient group where symptoms cannot always be reported clearly. Furthermore, a reliable assessment may be limited due to patient co-operation, consequently affecting reliability of the investigation and diagnosis from the referring GP.

This study examined the accuracy of GP paediatric referrals into the Manchester Royal Eye Hospital Orthoptic Department between the years 2013 and 2017.

The research aims were:

- To evaluate the accuracy of paediatric GP referrals (this included referrals from GP via community based optometrists) into the Orthoptic department at MREH
- To investigate potential barriers to referral accuracy

## Methodology

Trust approval was granted and no ethical approval was necessary due to this study being a retrospective case note ananlysis. This study meets the tenets of the Declaration of Helsinki. The research team adhered to General Data Protection Regulations.

For this analysis, a list of referrals to the Ophthalmology Department at MREH made between March 2013 and November 2017 was obtained and examined by orthoptists in the research team.

An accurate referral was defined as a GP appropriately referring a patient to the ophthalmology department with suspicions of a condition that was later confirmed following hospital consultation. This did not require the GP to offer a full diagnosis, but to identify features that matched the final diagnosis.

Inclusion criteria for referrals were:

- Referral made between January 2013 and December 2017
- First referral from a GP (including referrals from GP via community based optometrist). Re-referrals were excluded.
- Referral was for an orthoptic condition
- Patients < 18 years old

The following data were collected from each medical record:

- Referral source: Separated into GP and GP via community optometrist
- Reason for referral, taken from the free text section on the referral form
- Age of the patient at referral and when seen in clinic
- Final diagnosis made at MREH

The final diagnoses made at MREH were organised into the following diagnosis categories:

○ Strabismus
○ Reduced VA/refractive error/amblyopia
○ NAD (no apparent deviation, but also included family history of strabismus, parental concern or pseudostrabismus)
○ Nystagmus
○ Other

In order to allow for analysis of referral accuracy, each referral was assigned to one of the following codes:

1. Referral matched final diagnosis
2. Referal partially matched final diagnosis
3. Referral did not match final diagnosis
4. Referral did not match final diagnosis but incidental finding confirmed at hospital appointment that would have required referral

For data analysis, codes 1 and 2 were grouped together and named “matching”, and 3 and 4 were grouped together and named “non-matching”.

Refractive reasons for referral were deemed to match if the clinician recorded the same diagnosis as the referrer, regardless of prescription strength. Diagnoses of “reduced vision” were still included and classed as correct if the eventual diagnosis was not an orthoptic condition but did result in compromised vision, e.g. retinal dystrophy.

Statistical analysis was performed using SPSS V24. Comparisons of the categorical coding of outcomes were performed using the Chi-squared test.

## Results

A total of 1015 ophthalmology referrals were examined. Ninety-nine sets of medical records met the inclusion criteria. Mean age (±SD) at time of referral was 4.9 (±3.6) years.

### Accuracy of referrer

**Figure 1:**
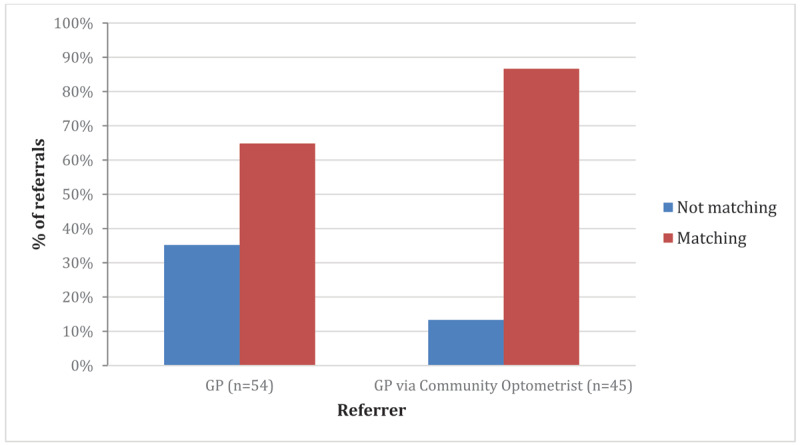
Does reason for referral match eventual diagnosis?

Fifty-four patients (55%) were referred directly from the GP. Forty-five (45%) patients had visited their own optometrist who completed an assessment, made a suggested diagnosis, and initiated a referral through the GP. ***Figure 1*** displays the percentages of matching referrals for GPs alone and for GP referral via community optometrist. When the referral accuracy was compared between GP-alone referrals and GP referrals via community optometrists, chi-squared analysis found a p value of 0.01.

### Accuracy of referrals

***Table 1*** shows the percentage of matching referrals for GPs alone, GPs when referral was via community optometrist, and total percentage of matching referrals.

**Table 1:** Accuracy of referrals by diagnosis.


CATEGORY	TOTAL % OF MATCHING REFERRALS	% OF MATCHING REFERRALS FROM GP ALONE	% OF MATCHING REFERRALS FROM GP VIA COMMUNITY OPTOMETRIST

Strabismus	77% (n = 35)	63% (n = 24)	90% (n = 11)

Reduced vision/amblyopia/refractive error	88% (n = 25)	50% (n = 2)	91% (n = 23)

NAD/pseudo strabismus	50% (n = 24)	45% (n = 20)	75% (n = 4)

Nystagmus	100% (n = 2)	100% (n = 1)	100% (n = 1)

Other	92% (n = 13)	100% (n = 7)	83% (n = 6)


### Impact of Age

***Table 2*** summarises the percentage of matching referrals for each age group in total as well as separated into GP-alone referral and GP referral via community optometrist.

**Table 2:** Proportion of referrals that matched their diagnosis by age.


AGE (YEARS)	% OF MATCHING REFERRALS		

	TOTAL	GP ALONE	GP VIA COMMUNITY OPTOMETRIST

0–2	61% (n = 33)	63% (n = 32)	0% (n = 1)

3–6	79% (n = 39)	69% (n = 16)	87% (n = 23)

7–13	85% (n = 27)	50% (n = 6)	90% (n = 21)


### Statistical results

Referrals were significantly more accurate when a community optometrist initiated the referral compared to a GP alone (p = 0.01). When comparing referral accuracy in relation to age, there was no significant difference between age groups (p = 0.06).

## Discussion

The data from this study showed that referrals coming from GPs via community optometrists were more accurate, but GP referrals alone were still reasonable. NAD was the least accurate of all the diagnostic categories at referral. An apparent trend of greater referral accuracy for older patients was observed, but this was not found to be statistically significant. Referrals from GP via community optometrist also showed a higher percentage of matches in the reduced vision category (see ***Table 1***), indicating that accuracy can be influenced by what the patient is initially referred for; however, the small sample size limits the validity of conclusions that can be drawn from these data. Optometrists are experts in diagnosing visual problems, and it is therefore logical that they are able to more accurately examine patients reporting reduced vision compared to GPs.

Appropriateness and accuracy of GP referrals varies from 39–90% across different professions (Elwyn et al. 1994; Blomgren et al. 2003; Ahmed et al. 2014; Gormley et al. 2003), but the accuracy rate in our study of 65% fits within the reported range. As anticipated, referrals to GP via community optometrists had a higher accuracy rate than GP referrals alone (87% and 65% respectively). A similar trend was reported by Davey et al. (2016) with accuracies of 67% for community optometrists and 57% for GP referrals for ophthalmological conditions. The higher accuracy of referrals from GP via optometrists in our study may be because orthoptic referrals were considered, while Davey et al. analysed general ophthalmology referrals. It is interesting to note that a matching referral rate of 77% for strabismus cases was seen, while Wang et al. (1990) found only 58% of cases with a correct diagnosis at referral. This could suggest GP referral accuracy for strabismus has been improving over the last 30 years. However it is difficult to directly compare to Wang’s findings as Wang did not look specifically at orthoptic referrals, instead looking at general ophthalmology referrals, which had a broader spectrum of diagnosis categories. Of these categories only three could be classified as orthoptic issues.

The least accurate of all the diagnostic categories was NAD, where half of the referrals had a false positive indication of strabismus. This may be due to difficulty in disproving strabismus without specific orthoptic training in detecting strabismus. For example, an infant with pseudo strabismus and epicanthic folds relies on accurate cover test, corneal reflection analysis, and binocular tests, rather than observations alone, to determine the correct diagnosis. Seventy-three percent of incorrect NAD cases were under 1 year of age, which would support this theory. The orthoptists also have a larger battery of tools available for orthoptic assessment and more experience within this area compared to referrers.

***Table 2*** appears to display a trend of referrals becoming more accurate the older a patient is at referral. This would seem logical because compliance and ability to report symptoms generally improves with age. However, this did not quite reach statistical significance (p = 0.06), which may be due to the small sample size. It was also observed that the older the child became, the more likely they were to attend the high street optometrist, as 97% of 0–2 year olds were assessed by GP alone whereas 78% of 7–13 year olds were assessed by a community optometrist who then initiated a GP referral. As older children are more able to communicate their signs and symptoms and more likely to attend the optometrist, this offers some explanation for why referrals via community optometrists are more accurate. No literature was found regarding the impact of patient age on accuracy of diagnosis.

There is a lack of consensus over what constitutes an accurate referral (O’Donnell et al. 2000). This may explain why there is such a range of perceived referral accuracy and appropriateness across different areas of medicine. It does seem unreasonable, however, to expect a GP to have 100% accuracy in any area of medicine, particularly one that is not their area of specialism. Rather, it is more appropriate to consider the role of the GP as one that will “formulate a problem list or definition that can open a gateway for further management” (RCGP 2007: para 8.18). In view of this and other available literature, the accuracy of GP referrals into the Orthoptic Department at MREH is considered acceptable by this study. There is, however, always room for improvement. While it is unknown whether additional training in a speciality increases or decreases a GP’s referral rate to that specialty (Davies et al. 2011), an educational approach to addressing GP referral quality and accuracy has been encouraged (Jones et al. 1990; Akbari et al. 2008). The study partners agree with this and believe that more bespoke training in orthoptics has the potential to improve referral accuracy. For example, training in strabismus detection using animations available on the internet could be an accessible and suitable method of orthoptic education for GPs.

While this study observed that the age of a patient could be a barrier to the accuracy of a referral, this trend did not reach statistical significance, likely because of the limited sample size when participants were divided into age groups. Additionally, another barrier could be the referrer’s understanding of orthoptic conditions. As a high false positive was found for strabismus referrals, this suggests GPs may benefit from orthoptic training in how to distinguish strabismus from pseudostrabismus. Jones et al. (1990) proposed a standard format of ophthalmology referral to ensure improved quality and accuracy. While a standard form may help to improve quality of GP referrals, it may not improve accuracy of referrals. This study suggests that virtual online educational packages covering strabismus detection be offered to GPs to improve their expertise in this area and therefore improve accuracy of diagnosis at referral. This would be beneficial as the package would be easily accessible to GPs without the need for travel or time constraints. A package may include videoed case examples, interactive learning opportunities, and a platform for GPs to reach out to orthoptists for tailored learning.

## Limitations

The eventual inclusion of only 10% of notes examined was significantly smaller than initially intended and this is a potential limitation. It also meant that when data were split to analyse particular factors, the samples were even smaller. The reason for this may be that MREH is a tertiary referral centre so many referrals come from other hospitals, specialties, emergency services, and social care, resulting in GPs being a minority of referral sources.

## Conclusion

An accurate referral was defined as a referral that identified features of a diagnosis that was later confirmed at the hospital appointment. Whilst not as accurate as community optometrists, the accuracy of GP referrals alone to the MREH Orthoptic Department is reasonable and comparable to other areas of medicine. Age may be a contributing factor towards the accuracy of paediatric referrals, with older pateints indicating better referral accuracy, but further research with a larger sample size would be needed to confirm this. A barrier to referral accuracy may be lack of GP training and orthoptic knowledge. This could be addressed with a virtual orthoptic training package for GPs.

This project was the first step towards improving GP accuracy in Orthoptic referrals by examining what current level of accuracy is. The next goal for the research team will be to design and implement the educational package suggested in this paper and distribute to local GPs and gather feedback on its efficacy.
